# Routine versus clinically indicated cerebrospinal fluid sampling for infection in external ventricular drains: a systematic review

**DOI:** 10.1007/s10072-026-09432-3

**Published:** 2026-09-26

**Authors:** Helbert de Oliveira Manduca Palmiero, Eberval Gadelha Figueiredo

**Affiliations:** Division of Neurosurgery, University of São Paulo Medical School, Dr. Enéas de Carvalho Aguiar Ave., 255, Room 5083, São Paulo, 05403-000 Brazil

**Keywords:** External ventricular drain, Cerebrospinal fluid, Ventriculostomy-related infection, Infection surveillance, Neurocritical care

## Abstract

**Background:**

External ventricular drains (EVDs) are commonly used in neurosurgery but carry a risk of ventriculostomy-related infection. Routine cerebrospinal fluid (CSF) sampling is commonly performed for surveillance, although its clinical value and potential risks are still uncertain.

**Methods:**

A systematic review was performed following PRISMA 2020 guidelines. PubMed was searched from inception to identify studies assessing CSF sampling strategies and/or clinical and laboratory screening for infection in patients with EVDs. Eligible studies included retrospective and prospective cohorts, case-control, and case series reporting infection-related outcomes. Data were extracted and categorized by causal relevance. Due to heterogeneity in studies, findings were summarized descriptively.

**Results:**

Fourteen studies were included, covering various study designs and sampling methods. Only 2 studies directly compared routine versus clinically indicated CSF sampling and found no benefit of routine daily sampling for detecting or reducing infections. Most studies were noncomparative and demonstrated the limited predictive value of routine clinical and laboratory parameters. Indirect evidence suggested that catheter duration, system manipulation, and patient-related factors were more strongly associated with infection risk than sampling frequency. Infection rates across studies ranged from 4.1% to 16.3%, with considerable variation in definitions and methodologies.

**Conclusion:**

Routine CSF sampling is not supported by high-quality evidence as a strategy to improve detection or prevent EVD-related infections. A selective, clinically guided approach may be more appropriate, with emphasis on reducing catheter manipulation and optimizing overall catheter management. Further prospective studies with standardized protocols are necessary to clarify the role of CSF sampling strategies.

## Introduction

External ventricular drainage (EVD) is a common neurosurgical intervention used for cerebrospinal fluid (CSF) diversion and intracranial pressure monitoring in patients with acute hydrocephalus, traumatic brain injury, subarachnoid hemorrhage, and other causes of intracranial hypertension [1, 2]. Despite its vital role, EVD placement and maintenance are associated with clinically significant risks, especially ventriculostomy-related infection, which contributes to increased morbidity, prolonged hospitalization, and higher healthcare costs [1, 3, 4]. Reported infection rates vary widely due to differences in diagnostic criteria, patient populations, and institutional practices [3, 5]. Several risk factors have been repeatedly identified, including the length of catheterization, frequency of CSF sampling or system manipulation, multiple catheter insertions, and underlying neurological conditions [3, 6].

Strategies to reduce EVD-related infections have concentrated on optimizing catheter management and minimizing modifiable risk factors. Preventive methods include antimicrobial-impregnated catheters, strict aseptic techniques, closed drainage systems, and the use of standardized care protocols [2, 7]. These bundles—containing coordinated interventions such as hand hygiene, barrier precautions, and controlled CSF sampling—have demonstrated reductions in infection risk, though results remain heterogeneous and the specific impact of individual components is unclear [3–5, 7]. Time-related analyses also indicate that infection risk varies throughout catheterization, with higher rates observed during specific periods of drainage, reinstating the importance of temporal risk assessment (Table 1) [8]. Additionally, in real-world practice, there is significant variability in EVD management, with inconsistent adherence to evidence-based protocols and ongoing gaps in provider knowledge and implementation [2, 9].

**Table 1:** Time-dependent incidence of meningoventriculitis associated with external ventricular and lumbar drainage reported by Scheithauer et al. [8]. The originally reported statistics are summarized; the per-day infection incidence was highest between days 4 and 9, with 82% of cases occurring within the first 12 days

Parameter	Reported value
Study design	Prospective observational cohort over 18 months (2007–2008)
Population	210 patients with an external ventricular (EVD) or lumbar (LD) drain
Meningoventriculitis (MV) cases	34 total (17 EVD, 17 LD)
Infection rate	7.5 per 1000 EVD-days; 24.7 per 1000 LD-days
Temporal distribution	82% (28/34) of cases within the first 12 days; incidence highest on days 4–9
Effect of drainage duration	Longer drainage (> 5 and > 9 days) associated with lower daily MV risk (*p* = 0.03; *p* < 0.001)
Independent risk factors	Lumbar vs. ventricular drain (OR 2.3), previous MV (OR 7.0), neoplasm (OR 11.6)

Beyond bundled care, several device- and technique-related factors modulate this baseline risk. Antimicrobial- and silver-impregnated catheters reduce infection risk compared with standard catheters. A randomized trial reported a reduction from 21.4% to 12.3% with silver-impregnated drains [10], and a subcutaneous tunneling length greater than 5 cm from the burr hole to the skin exit site is associated with significantly lower infection rates [11]. Clinical confounders, including cerebrospinal fluid leakage and subarachnoid hemorrhage, further increase risk [3, 6]. Notably, current evidence-based consensus guidance from the Neurocritical Care Society recommends against routine CSF sampling and advises obtaining CSF only when clinically indicated [12]; however, adherence in practice remains variable, and the evidence underpinning this recommendation had not been systematically synthesized, which is the specific gap this review addresses.

The diagnosis of EVD-related infection remains uncertain, as clinical signs and routine CSF laboratory parameters often lack specificity in the context of underlying neurological disease [4, 6]. Although microbiological culture remains the reference standard, results are delayed, and routine CSF surveillance continues to be widely practiced despite conflicting evidence regarding its diagnostic value and risk of EVD system contamination [4]. Given these uncertainties, the purpose of this systematic review was to evaluate whether routine CSF sampling is associated with ventriculostomy-related infection and to determine whether clinical and laboratory parameters can reliably guide infection detection without reliance on scheduled sampling.

## Methods

A systematic review was conducted in accordance with the PRISMA 2020 statement [13]. PubMed/MEDLINE was searched from its inception to the date of the final search for studies in English that assessed cerebrospinal fluid (CSF) sampling methods and/or clinical and laboratory screening for infection in patients with external ventricular drains (EVDs). The search combined controlled vocabulary and free-text terms related to *external ventricular drain*,* EVD*,* ventriculostomy*,* cerebrospinal fluid*,* CSF*,* sampling*,* culture*,* surveillance*,* monitoring*,* routine*,* scheduled*,* frequent*,* daily*,* clinically indicated*,* selective*,* on-demand*,* ventriculitis*,* infection*,* ventriculostomy-related infection*,* ventriculostomy-associated infection*,* retrospective*,* prospective*,* observational*,* cohort*,* and case series*.

PubMed/MEDLINE was selected as the primary bibliographic source because it offers the most comprehensive indexing of the neurosurgical and neurocritical-care literature relevant to EVD management and CSF sampling. Reliance on a single database is acknowledged as a limitation (see Limitations). This review was not prospectively registered in PROSPERO or the Open Science Framework.

The complete PubMed search string was: (“external ventricular drain*” OR EVD OR ventriculostomy) AND (“cerebrospinal fluid” OR CSF) AND (sampling OR culture OR surveillance OR monitoring) AND (routine OR scheduled OR frequent OR daily OR “clinically indicated” OR selective OR on-demand) AND (ventriculitis OR infection OR “ventriculostomy-related infection” OR “ventriculostomy-associated infection”).

Methodological quality was assessed using the Newcastle-Ottawa Scale (NOS) for cohort and case-control studies [14]. The selection, comparability (confounding), and outcome/exposure ascertainment domains were summarized into an overall risk-of-bias judgment (Table 2).

**Table 2:** Characteristics of included studies and main findings. Routine CSF sampling indicates scheduled sampling independent of clinical suspicion; clinically indicated sampling refers to sampling based on clinical or laboratory findings. Infection rates are reported as proportions unless otherwise specified. Hussein et al. [15] reported a mixed cohort of 232 patients with 437 devices (212 EVDs, 92 lumbar drains, and 133 ICP monitors); the reported infection figure is therefore not EVD-exclusive and is not included in the pooled EVD-specific infection-rate range. Thompson et al. [16] was a case-control study; *N* = 83 denotes infected cases compared with 249 controls, so no single study-level infection rate is available. Abbreviations: *EVD* external ventricular drain, *CSF* cerebrospinal fluid, *ICU* intensive care unit, *TBI* traumatic brain injury, *SAH* subarachnoid hemorrhage, *aSAH* aneurysmal subarachnoid hemorrhage, *ICH* intracerebral hemorrhage, *IVH* intraventricular hemorrhage, *ICP* intracranial pressure, *WBC* white blood cell count, *CRP* C-reactive protein, *GramPos* gram-positive, *GramNeg* gram-negative, *CoagNeg* coagulase-negative, *NR* not reported

Study (Year)	*N*	Pop.	EVD Indication	Duration of EVD (Days)	Catheter	Routine CSF Sampling	EVD Handling	Definition of Infection	Infection Rate	Time to Infection (Days)	Micro	Clinical Signs	Lab	Association Routine Sampling-Infection	Additional Findings
Hader (2000) [17]	157	Peds (ICU)	Severe TBI 41%, tumor with hydrocephalus 27.4%, elective ICP monitoring 13.4%	Mean 5.6	Not specified	Daily	Sterile	Culture + Clinical	6% (7/157)	NR	GramPos cocci (S. epidermidis, S. aureus)	Fever; neuro deter	Blood WBC	Routine sampling not beneficial vs. clinically indicated sampling	Routine cultures not earlier than clinical assessment
Arabi (2005) [18]	84	Adults (ICU)	TBI 63%, brain tumors 14%, ICH 21%	Mean 7 (infected cath 8 ± 6 vs. not infected 5 ± 3)	Not specified	Comparative (Daily vs. Clinical, protocol change)	Protocol	Culture + Clinical	14% (12/84)	Median 5 (2–8)	GramPos cocci and GramNeg bacilli	Fever; neuro deter; meningeal signs; local signs	CSF (cell count, glucose, protein)	Routine sampling not beneficial vs. clinically indicated sampling	Daily surveillance not superior to clinically indicated sampling
Bota (2005) [19]	638	Adults (ICU)	Severe TBI, aSAH, ICH, IVH, brain tumors	Median 9–15	Standard (non-antimicrobial)	Daily	Protocol	Culture + Clinical	9% (58/638)	Median 9	GramPos cocci (S. epidermidis, S. aureus)	Fever; meningeal signs	CSF (cell count, glucose, protein, lactate), blood WBC	No direct assessment	No direct sampling comparison; duration/case-mix factors predominated
Muttaiyah (2008) [20]	60	Mixed (Ward)	SAH 41%, ICH 17%, tumor 18%, shunt malfunction 12%, other hydrocephalus 12%	Median 6 (1–23)	Not specified	Daily	Sterile	Culture + Clinical	15% (9/60)	Median 10 (1–16)	CoagNeg staphylococci, S. epidermidis	Fever; neuro deter	CSF (cell count, glucose, protein)	Clinical/lab screening study; no direct assessment	Clinical signs alone did not predict infection
Mounier (2015) [21]	449	Adults (ICU)	Hydrocephalus 94%, mainly secondary to aSAH	Median 15	Antibiotic-impregnated	Daily	Protocol	Culture + Clinical	7.1% (32/449)	Median 12	GramPos (S. epidermidis) and GramNeg (P. aeruginosa, K. pneumoniae)	Fever; neuro deter	CSF (cell count, glucose, protein), blood WBC	Clinical/lab screening study; no direct assessment	Serial trends, not single markers, supported diagnosis
Hariri (2017) [22]	123	Adults (ICU)	TBI most common; SAH, IVH, cerebral edema, hydrocephalus	NR	Standard (non-antimicrobial)	Three times weekly (Intermittent schedule)	Protocol	Lozier-based (modified)	8.8% (11/123)	NR	CoagNeg staphylococci, S. epidermidis	Fever; neuro deter	CSF (cell count, glucose, protein), blood WBC	Clinical/lab screening study; no direct assessment	No single clinical or laboratory predictor was reliable
Hussein (2019) [15]	232	Adults (ICU)	Hydrocephalus/increased ICP 28.4%, TBI 38.2%, ICH 12.6%, SAH 10.3%, infection 6.6%, CSF leak 3.9%	NR	Not specified	Variable (Not standardized)	Bundle	CDC/NHSN-based	22.8% (53/232, episodes)	NR	GramNeg bacilli (A. baumannii, K. pneumoniae, P. aeruginosa)	NR	CSF (cell count), other serum inflammatory markers	Indirect evidence: manipulation/access associated with infection risk	Drain manipulation and duration associated with infection
Nisson (2019) [23]	180	Mixed (ICU)	ICH most common 61.5%, tumor 16.8%, shunt malfunction 3.9%	NR	Antibiotic-impregnated	Twice weekly	NR	CDC/NHSN-based	5.6% (10/180)	NR	NR	Systemic signs	Blood WBC	Clinical/lab screening study; no direct assessment	Peripheral WBC may support targeted screening
Thompson (2018) [16]	83	Mixed (NR)	Mixed (Not specified)	Median 11–14 (> 10, risk of infection)	Not specified	Variable (Not standardized)	Protocol	Lozier-based (modified)	NR (83 infected cases vs. 249 controls)	< 30d	NR	Fever; meningeal signs	CSF (cell count)	Routine sampling not associated with higher infection risk	Recurrent sampling not associated with increased infection
Champey (2018) [24]	462	Adults (ICU)	SAH most common, ICH, TBI	Median 11–14	Standard (non-antimicrobial)	Comparative (Daily vs. Clinical)	Bundle	Lozier-based	1.4–9.2%	NR	NR	Fever; neuro deter	CSF (cell count, glucose, protein), blood WBC, CRP	Lower infection observed with routine sampling, but confounded	Lower infection with daily sampling center, but heavily confounded by bundle differences
Ponnambath (2024) [25]	211	Mixed (ICU)	Tumor excision most common, 58.2%	> 7d, risk of infection	Not specified	Variable/Periodic	Bundle	IDSA-based	7.1% (15/211)	NR	GramNeg bacilli (K. pneumoniae, A. baumannii, P. aeruginosa)	Fever; neuro deter	CSF (cell count, glucose, protein)	Routine sampling associated with higher infection risk	Periodic surveillance sampling associated with infection
Roujansky (2024) [26]	227	Adults (ICU)	SAH 85%, TBI 15%	Median 12	Mixed (Standard and Antibiotic-Impregnated)	Daily	Protocol	Lozier-based (modified)	4.8% (11/227)	Median 16	CoagNeg staphylococci, S. aureus	Fever; neuro deter	CSF (cell count, glucose, protein), blood WBC	Indirect evidence: manipulation/access associated with infection risk	Manipulation burden and duration associated with infection
Stahlberg (2025) [27]	1828	Adults (ICU)	Mixed (Not specified)	NR	Standard (non-antimicrobial)	Twice weekly+Clinically indicated	Protocol	Culture + Clinical	4.1% (75/1828)	NR	NR	Fever; neuro deter	CSF (cell count, glucose, protein, lactate), blood WBC	Clinical/lab screening study; no direct assessment	Surveillance markers poorly distinguished true from suspected infection
Alakmeh (2026) [28]	387	Adults (ICU)	aSAH most common	NR	Antibiotic-impregnated	Twice weekly+Clinically indicated	Protocol	Composite (study-defined)	16.3% (63/387)	NR	NR	Fever; neuro deter; meningeal signs	CSF (cell count), blood WBC, CRP, procalcitonin	No direct assessment	No direct sampling comparison; exchange and pathology drove risk

Two reviewers independently screened titles and abstracts, followed by full-text review of studies that appeared potentially eligible. Disagreements were resolved through consensus. Studies were considered eligible if they reported original clinical data on CSF sampling and/or laboratory screening in EVD-treated patients and provided extractable infection-related outcomes from either retrospective or prospective cohort studies or case series. Case reports, review articles, editorials, commentaries, conference abstracts without full text, and unrelated studies were excluded. Studies primarily focused on permanent ventricular shunts or lumbar drains were excluded from the comparative synthesis.

Data were extracted using a standardized form. Variables of interest included publication year, study design, sample size, patient population, indication for EVD placement, catheter type, CSF sampling strategy and frequency, EVD handling protocol, definition of infection, infection rate, time to infection, causative microorganisms, clinical signs, laboratory parameters, and the reported relationship between routine CSF sampling and infection. Studies were then qualitatively categorized based on causal relevance as direct comparisons, indirect comparisons, or diagnostic/non-comparative studies.

**Fig. 1:**
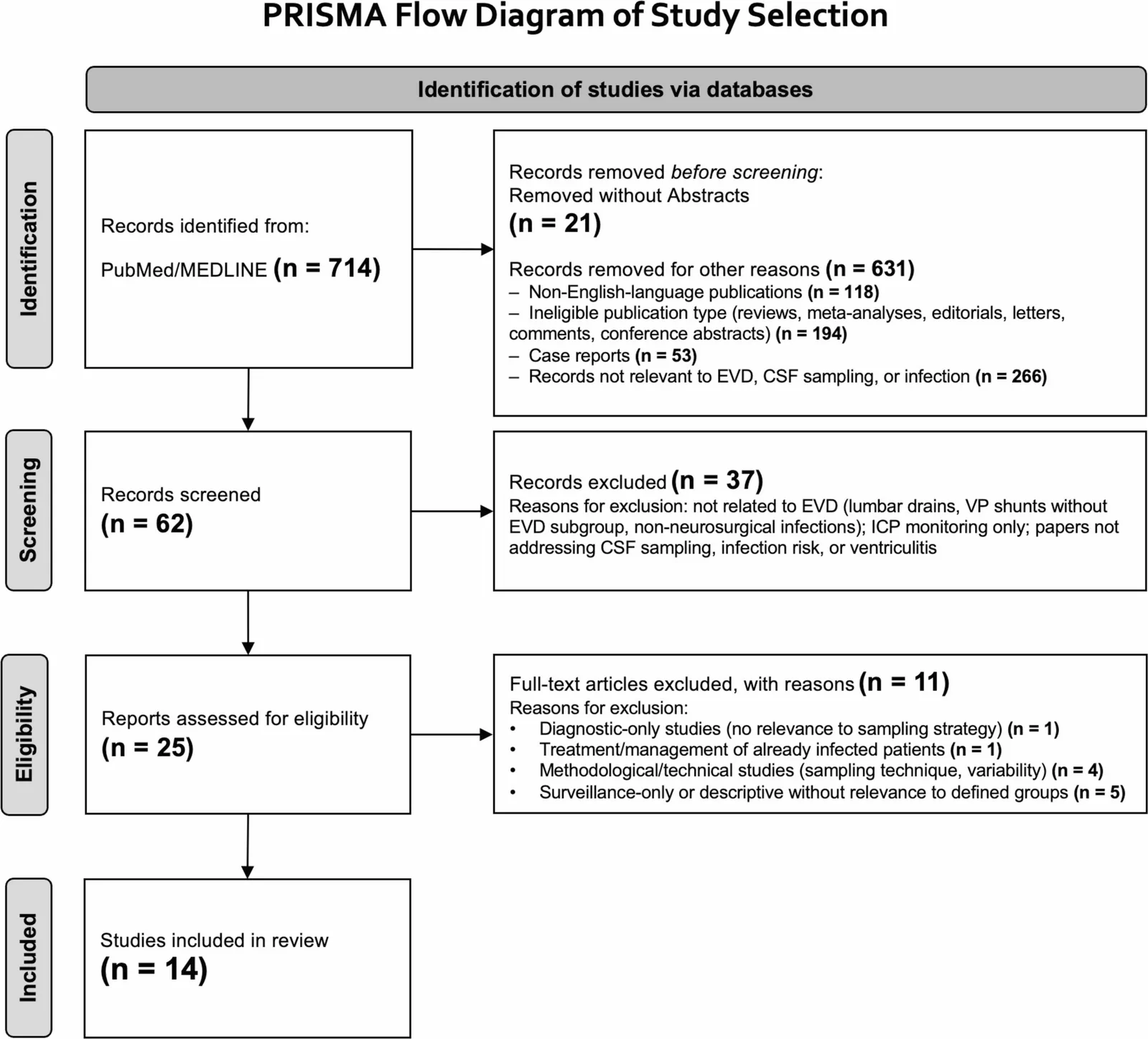
PRISMA flow diagram illustrating study identification, screening, eligibility assessment, and inclusion process for the systematic review, resulting in 14 studies included in the synthesis

A total of 714 records were identified through database searching. After screening and assessing full-text eligibility, 14 studies were included in the qualitative synthesis (Fig. 1), with records removed before screening categorized as those without an abstract, non-English-language publications, ineligible publication types (reviews, meta-analyses, editorials, letters, comments, and conference abstracts), case reports, and records not relevant to EVD, CSF sampling, or infection. Due to significant heterogeneity in study design, definitions of infection, sampling protocols, and outcome reporting, a formal meta-analysis was not conducted. Instead, the comparative findings were summarized descriptively.

This review was solely based on published data. Therefore, approval from an institutional review board and informed consent were not necessary.

## Results

The literature search identified 714 records, of which 14 studies met the eligibility criteria and were included in the qualitative synthesis (Fig. 1). The included studies were published between 2000 and 2026 and included retrospective cohorts, prospective cohorts, case-control studies, and one ambispective cohort. Sample sizes ranged from 60 to 1828 patients. Most studies were conducted in adult intensive care unit populations, although pediatric and mixed populations were also represented. Reported infection rates varied considerably, from 4.1% to 16.3% when expressed as study-level proportions, reflecting differences in case mix, surveillance strategies, and infection definitions across studies (Table 2).

Study methods were heterogeneous. Routine CSF surveillance ranged from daily sampling to intermittent scheduled sampling, twice-weekly surveillance, hybrid strategies combining scheduled and clinically indicated sampling, and variable or nonstandardized sampling practices. Direct comparisons between routine and clinically indicated sampling were available in only 2 studies (Hader et al. [17], Arabi et al. [18]). Most studies instead evaluated diagnostic performance under a single surveillance protocol or assessed sampling frequency indirectly through manipulation burden or recurrent sampling exposure. Definitions of infection also varied, including culture-plus-clinical criteria, modified Lozier-based criteria, CDC/NHSN-based definitions, IDSA-based definitions, and study-specific composite definitions (Table 2).

The risk-of-bias assessment revealed that most studies were level III observational designs, with 3 prospective cohort studies classified as level II evidence. Overall, the risk of bias was moderate in 4 studies, moderate-high in most of the remainder, and high in 1 multicenter retrospective cohort study. Across all studies, the principal methodological concerns included moderate limitations in exposure and outcome assessment and a substantial risk of confounding, especially in studies evaluating bundled interventions or nonrandomized differences in surveillance practice (Table 3).

**Table 3:** Risk of bias assessment of included studies. Risk of bias was assessed across domains, including selection bias, exposure/outcome assessment, and confounding factors, using the Newcastle-Ottawa Scale (NOS) for cohort and case-control studies. The selection, comparability (confounding), and outcome/exposure ascertainment domains were summarized into an overall judgment. The overall risk was classified as low, moderate, moderate-high, or high. The level of evidence was graded per the Oxford Center for Evidence-Based Medicine hierarchy (II = prospective cohort; III = retrospective cohort or case-control)

Study	Design (Level of Evidence)	Selection Bias	Exposure/Outcome Assessment	Confounding	Overall Risk of Bias
Hader [17]	Retrospective cohort (III)	Moderate	Moderate	Moderate	**Moderate**
Arabi [18]	Retrospective cohort (III)	Moderate	Moderate	High	**Mod–High**
Bota [19]	Retrospective cohort (III)	Moderate	Moderate	High	**Mod–High**
Muttaiyah [20]	Retrospective cohort (III)	Moderate	Moderate	High	**Mod–High**
Mounier [21]	Retrospective cohort (III)	Moderate	Moderate	High	**Mod–High**
Hariri [22]	Retrospective cohort (III)	Moderate	Moderate	High	**Mod–High**
Hussein [15]	Prospective cohort (II)	Low	Moderate	Moderate	**Moderate**
Nisson [23]	Case-control (III)	Moderate	Moderate	High	**Mod–High**
Thompson [16]	Case-control (III)	Moderate	Moderate	High	**Mod–High**
Champey [24]	Retrospective multicenter cohort (III)	Moderate	Moderate	High	**High**
Ponnambath [25]	Prospective cohort (II)	Moderate	Moderate	High	**Mod–High**
Roujansky [26]	Prospective cohort (II)	Low	Moderate	Moderate	**Moderate**
Ståhlberg [27]	Retrospective cohort (III)	Moderate	Moderate	High	**Mod–High**
Alakmeh [28]	Ambispective cohort (III)	Low	Moderate	Moderate	**Moderate**

Only 2 studies directly compared routine sampling with clinically indicated sampling (Hader et al. [17], Arabi et al. [18]). In both, routine daily CSF sampling did not demonstrate a clear advantage. Hader et al. [17] found that routine daily cultures did not detect infection before clinical signs appeared, and Arabi et al. [18] reported no reduction in infection with routine surveillance cultures compared to cultures obtained when clinically indicated (Table 4). These 2 studies provided the most relevant causal evidence in the review, and both were neutral regarding the benefit of routine sampling.

**Table 4:** Qualitative synthesis of evidence regarding routine CSF sampling and EVD-related infection. Studies are categorized by causal relevance: direct comparisons (routine vs. clinically indicated sampling), indirect comparisons (sampling frequency or device manipulation), and diagnostic/non-comparative designs. Routine sampling denotes scheduled CSF collection independent of clinical suspicion. *Conclusions need to be interpreted considering heterogeneity and potential confounding*

Study	Sampling comparison type	Routine sampling exposure	Main conclusion about routine sampling
Hader [17]	**Direct comparison**	Daily vs. clinically indicated	Routine sampling does not improve early detection of infection
Arabi [18]	**Direct comparison**	Daily vs. clinically indicated	No reduction in infection with routine sampling compared to clinically indicated
Bota [19]	Non-comparative	Daily routine sampling	No conclusion regarding sampling; infection driven by duration and patient factors
Muttaiyah [20]	Non-comparative (diagnostic)	Daily routine sampling	Routine sampling does not reliably predict infection
Mounier [21]	Non-comparative (diagnostic)	Daily routine sampling	Infection diagnosis depends on dynamic trends, not sampling frequency
Hariri [22]	Non-comparative (diagnostic)	Intermittent scheduled sampling	Routine surveillance parameters lack predictive reliability
Hussein [15]	**Indirect comparison** (manipulation exposure)	Sampling frequency not standardized	Increased device manipulation associated with infection risk (indirect signal)
Nisson [23]	Non-comparative (diagnostic)	Twice weekly routine sampling	Routine surveillance limited; alternative markers (WBC) may assist detection
Thompson [16]	**Indirect comparison** (sampling frequency exposure)	Variable/recurrent sampling	Increased sampling frequency not associated with higher infection risk
Champey [24]	**Indirect comparison** (bundle-confounded)	Daily vs. selective sampling	Lower infection observed with routine sampling, but confounded by care bundle
Ponnambath [25]	**Indirect comparison** (risk factor analysis)	Periodic/variable sampling	Routine/periodic sampling associated with infection risk, not causally isolated
Roujansky [26]	Non-comparative	Daily routine sampling	Infection associated with clinical context and duration, not sampling strategy
Ståhlberg [27]	Non-comparative (diagnostic)	Twice weekly + clinically indicated sampling	Routine surveillance poorly discriminates true infection and may lead to overtreatment
Alakmeh [28]	Non-comparative	Twice weekly + clinically indicated sampling	Infection driven by patient and procedural factors; no sampling effect demonstrated

Most of the remaining studies were noncomparative and focused on diagnostic utility rather than causal effects of sampling frequency. Daily routine surveillance was used in several retrospective or prospective cohorts, yet these studies consistently suggested that routine clinical or laboratory screening alone had limited predictive value. Muttaiyah et al. [20] found that temperature and Glasgow Coma Scale did not allow early prediction of infection. Mounier et al. [21] reported that diagnosis depended on serial clinical and CSF trends rather than isolated measurements. Hariri et al. [22] likewise found no single clinical or laboratory predictor to be reliable under an intermittent scheduled surveillance strategy. In the largest cohort, Ståhlberg et al. [27] showed that twice-weekly plus clinically indicated surveillance poorly distinguished true infection from suspected infection and was associated with substantial overtreatment. Nisson et al. [23] suggested that peripheral white blood cell count might support screening, but did not demonstrate a sampling-frequency effect. Alakmeh et al. [28] similarly found that infection was driven by patient and procedural factors rather than a measurable effect of the surveillance schedule (Table 4).

Indirect evidence regarding manipulation or recurrent sampling showed mixed but understandable patterns. Hussein et al. [15] identified drain opening and manipulation as independent risk factors for infection, supporting an indirect signal that additional system access may be harmful. Ponnambath et al. [25] also identified periodic surveillance CSF sampling as a risk factor in multivariable analysis, although this finding emerged in the context of broader bundle development and does not isolate a causal effect of sampling itself. In contrast, Thompson et al. [16] found that recurrent sampling was not associated with a higher infection risk in a case-control analysis. Champey et al. [24] reported lower infection rates at the center performing daily sampling, but this result was inseparable from other differences in the care bundle and thus remained highly confounded (Table 4).

**Fig. 2:**
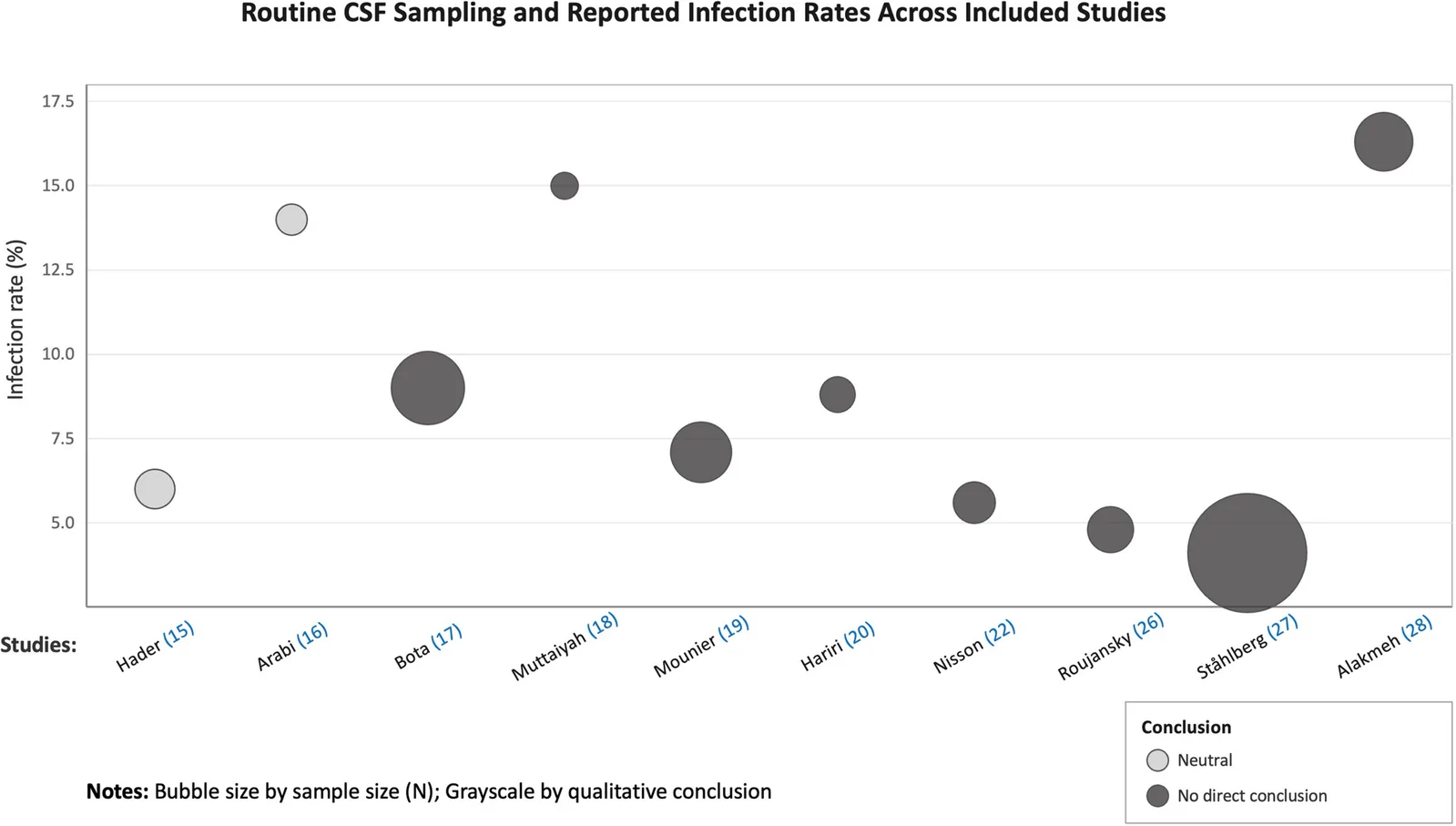
Routine CSF sampling and reported infection rates across included studies. Each bubble represents an individual study, with the y-axis showing the reported ventriculostomy-related infection rate (%). The size of each bubble reflects the sample size (N). Grayscale shading indicates study-level qualitative conclusions about routine cerebrospinal fluid (CSF) sampling: light gray = neutral (no observed benefit) and dark gray = no definitive conclusion. Interpretations are based on study-reported results and should be considered in the context of heterogeneity in study design, infection definitions, and potential confounding factors. Note: studies without a single EVD-specific study-level infection rate were not plotted, including Thompson et al.‘s case-control design [16] (83 infected cases vs. 249 controls), Hussein et al.‘s mixed CSF-drain and ICP-monitoring cohort [15], multicenter studies reporting only site-specific rates [24], and bundle-development cohorts [25]

Infection rates across studies ranged from 4.1% to 16.3%, with considerable variation in definitions, study design, and patient populations. One additional cohort (Hussein et al. [15]) reported a higher figure (22.8%), but because it combined ventricular drains with lumbar drains and ICP monitors, it is not directly comparable as an EVD-specific rate (Table 2). Study-level comparisons did not demonstrate a consistent association between routine CSF sampling practices and infection rates, whether for improved infection detection or increased risk from drain manipulation. Instead, indirect evidence more consistently identified catheter duration, system manipulation (Fig. 2), and patient-related factors as contributors to infection risk. These findings should be interpreted cautiously given the predominance of noncomparative study designs and heterogeneity across studies. Overall, the bubble plot shows that studies reporting an EVD-specific study-level infection rate had neutral or inconclusive findings; signals suggesting possible harm arose from studies not represented in the plot (a case-control design and a mixed-device cohort), and none provided unconfounded direct comparative evidence (Fig. 2).

## Discussion

This systematic review found limited direct evidence that routine cerebrospinal fluid (CSF) sampling improves the detection or prevention of external ventricular drain (EVD)–related infection. Only 2 studies directly compared scheduled surveillance with clinically indicated sampling, and neither demonstrated a clear benefit from routine daily cultures [17, 18]. The remainder of the literature mainly consisted of noncomparative diagnostic studies and indirect observational analyses, which consistently suggested that infection risk is more strongly associated with catheter duration, CSF leak, drain manipulation, repeated catheter insertion or exchange, and concurrent infection than with any specific surveillance schedule itself (Tables 1 and 3). In practical terms, the available evidence does not support routine, scheduled CSF sampling as an evidence-based strategy to improve outcomes, and it raises concerns that repeated access to the drainage system may itself increase risk in some settings.

The most important finding of the review is the discrepancy between the continued use of routine sampling in clinical practice and the limited evidence supporting it. Several studies employing daily or intermittent routine protocols demonstrated that standard clinical and laboratory markers lack sufficient predictive value to reliably detect infection before obvious clinical signs appear [20–23, 27]. Even in studies aimed at improving surveillance, positive microbiological results often required interpretation alongside changing CSF trends, systemic inflammatory markers, and the underlying neurological condition, rather than serving as standalone screening tools [21, 27]. This is clinically important because patients with subarachnoid hemorrhage, intraventricular hemorrhage, traumatic brain injury, or recent craniotomy frequently exhibit fever, pleocytosis, altered mental status, and abnormal CSF biochemistry without actual ventriculostomy-related infection. Therefore, the diagnostic problem is not just about sampling frequency but also concerns low specificity in a biologically noisy population.

The indirect evidence should also be interpreted cautiously. Studies linking drain opening, recurrent access, or periodic surveillance to higher infection rates suggest a biologically plausible harm signal, but they do not isolate routine CSF sampling from the broader context of catheter handling [15, 25]. Conversely, Thompson et al. found no association between recurrent sampling and infection, underscoring how much these findings may depend on local protocols, aseptic techniques, case mix, and confounding factors like drainage duration or illness severity [16]. Champey et al. reported lower infection rates in the center performing daily sampling, but this was inseparable from differences in the care bundle and therefore cannot be attributed to sampling alone [24]. Overall, the literature does not confirm that scheduled sampling is inherently harmful, but it also does not demonstrate that it is beneficial. For that reason, the most defensible interpretation is that routine sampling should not be considered a proven preventive or diagnostic measure independent of the larger catheter-management environment.

Another important implication of this review is that infection prevention and infection detection should be viewed separately. Measures such as antimicrobial-impregnated catheters, sterile insertion techniques, bundled maintenance practices, and minimizing unnecessary system manipulation may lower ventriculostomy-related infection risk, but these interventions focus on prevention rather than surveillance [1–9]. In contrast, scheduled CSF sampling is primarily a surveillance method, and the evidence presented here indicates that its clinical benefit is limited when used without discretion. The stronger and more consistent associations across studies involved exposure-related variables—catheter dwell time, leaks, exchanges, and access burden—more than whether CSF was sampled daily, intermittently, or twice weekly. This distinction is important because it shifts the focus from routine culturing to disciplined catheter management.

### Clinical neurosurgical practice

**Fig. 3:**
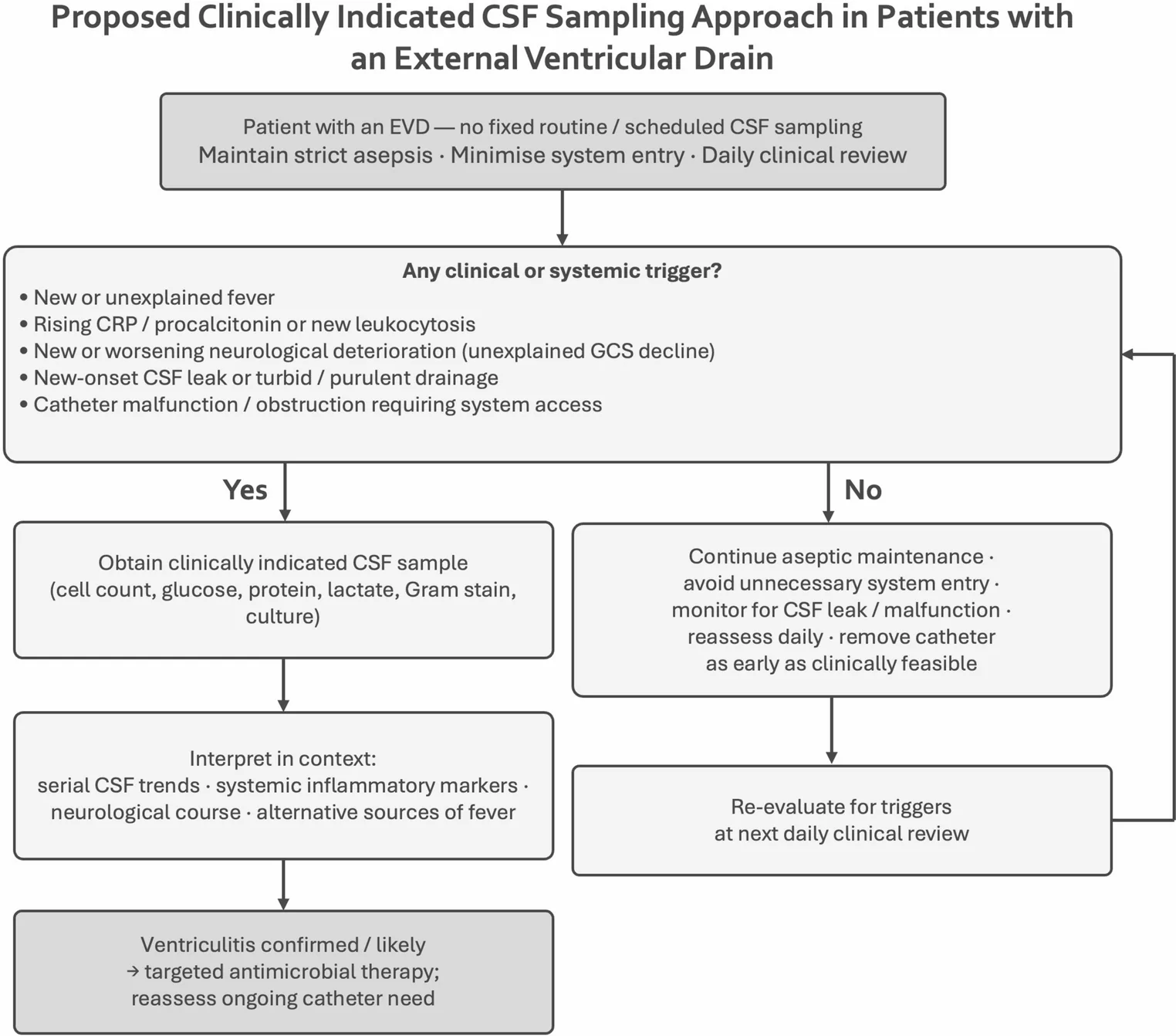
Proposed decision pathway for clinically indicated CSF sampling in patients with an external ventricular drain. In the absence of routine scheduled sampling, a CSF sample is obtained when a clinical or systemic trigger is present (new or unexplained fever; rising CRP/procalcitonin or leukocytosis; new or worsening neurological deterioration; new-onset CSF leak or turbid/purulent drainage; catheter malfunction requiring access). Results are interpreted alongside serial CSF and systemic trends and the neurological course

For neurosurgical practice, these findings support a more selective approach to CSF sampling. Routine scheduled cultures may be appropriate in centers that have incorporated them into a tightly controlled protocol, but the current review does not justify them as a universal standard. Instead, clinicians should prioritize strict aseptic handling, minimizing system entry, promptly recognizing CSF leaks or malfunctions, and removing the catheter early when clinically feasible. When infection is suspected, CSF results should be interpreted alongside the temporal pattern of CSF indices, systemic inflammatory markers, neurological progress, and other potential sources of fever or inflammation. In this context, clinically indicated sampling appears more justifiable than indiscriminate surveillance, especially in patients where repeated system opening can be avoided without compromising care. However, selective sampling should not cause delays; it should mean targeted sampling guided by evolving clinical and biochemical signs rather than on schedule alone. To translate these findings into practice, a simple decision pathway is proposed for clinically indicated CSF sampling, outlining the clinical and systemic triggers for obtaining a CSF sample and the subsequent steps for interpretation and management (Fig. 3).

### Limitations

The evidence base was highly heterogeneous with respect to patient population, EVD indication, catheter technology, antimicrobial prophylaxis, infection definition, and surveillance protocol, precluding a formal meta-analysis. Most included studies were observational and had a moderate to high risk of bias, with confounding the main methodological limitation (Table 2). Only 2 studies offered direct comparisons of routine versus clinically indicated sampling, and both were retrospective [17, 18]. Some studies assessed sampling frequency indirectly through measures of manipulation burden or recurrent access, limiting causal interpretations. Outcome definitions varied across studies, from culture-based criteria to combined clinical and laboratory criteria, which likely contributed to the wide range of reported infection rates. In particular, rates derived from culture positivity or catheter colonization are systematically higher than those restricted to clinically and microbiologically confirmed ventriculitis, so the observed variation reflects definitional and case-mix differences more than true differences in underlying risk. Additionally, publication bias and institutional practice bias are possible, especially since surveillance strategies are often part of larger local protocols rather than tested as isolated exposures. These limitations argue for caution in drawing definitive recommendations, but they do not diminish the central observation that high-quality evidence supporting routine CSF surveillance remains limited. The search was also restricted to a single database (PubMed/MEDLINE), which, despite its comprehensive coverage of the relevant literature, may have missed studies indexed only elsewhere and represents a potential source of selection bias. The review was not prospectively registered.

## Conclusion

Routine cerebrospinal fluid (CSF) sampling in patients with external ventricular drains is not supported by high-quality evidence as a method to improve detection or prevent ventriculostomy-related infections. The only existing direct comparative studies demonstrated no benefit of scheduled sampling over clinically indicated approaches. Meanwhile, broader noncomparative and indirect evidence consistently indicate limited diagnostic value and emphasize that catheter duration, system manipulation, and patient-related factors are more important in influencing infection risk. These findings suggest that routine CSF surveillance should not be considered standard practice regardless of overall catheter management, and a more targeted, clinically guided approach may be more suitable in many settings. Future prospective studies with standardized infection definitions and controlled catheter-care protocols are necessary to clarify the role of CSF sampling strategies and to determine whether targeted surveillance can optimize detection while minimizing unnecessary system access and potential harm.
